# Multimodality Imaging Approach in Diagnosis and Follow-Up of Aortic Coarctation in Adulthood

**DOI:** 10.3390/jcm15030949

**Published:** 2026-01-24

**Authors:** Lucia La Mura, Luigi Mannacio, Federica Illuminato, Marco Ferrone, Maria Lembo, Saverio D’Elia, Carmine Izzo, Ciro Santoro, Raffaele Izzo

**Affiliations:** 1 Department of Advanced Biomedical Sciences, “Federico II” University of Naples, 80131 Naples, Italy; luigimannacio@gmail.com (L.M.); maria.lembo@unina.it (M.L.); raffaele.izzo@unina.it (R.I.); 2 “Ferdinando Veneziale” Hospital, Università Degli Studi Del Molise, 86100 Campobasso, Italy; feilluminato@gmail.com; 3 Catheterization Laboratory, Division of Cardiology, Montevergine Clinic, 83013 Mercogliano, Italy; marco.ferrone1@gmail.com; 4 Cardiology Unit, AOU Luigi Vanvitelli, 80138 Naples, Italy; saveriodelia85@gmail.com; 5 Department of Advanced Medical and Surgical Sciences, University of Campania “Luigi Vanvitelli”, Piazza Luigi Miraglia, 2, 80138 Naples, Italy; 6 Department of Medicine, Surgery and Dentistry, University of Salerno, Via Salvador Allende, 84081 Baronissi, Italy; cizzo@unisa.it; 7 Department of Pharmacy, Health and Nutritional Sciences, University of Calabria, Rende (CS), 87036 Arcavacata, Italy; ciro.santoro@unical.it

**Keywords:** aortic coarctation, CTA, CMR, CE-MRA, TTE, CT angiography, multimodality imaging, echocardiography, cardiovascular MRI, adult aortic coarctation

## Abstract

Aortic coarctation (CoA) comprises local narrowing of the aortic lumen, which is located at the level of the isthmus in 95% of patients and accounts for 5 to 8% of live births with congenital heart disease. It can be associated with other congenital defects, such as a bicuspid aortic valve (BAV), and in adulthood should be considered a secondary cause of hypertension in patients younger than 40 years, particularly in the presence of severe or resistant hypertension, in accordance with current hypertension guidelines. A correct diagnosis is necessary for the proper assessment and management of these patients. A multimodality imaging approach using ultrasound, computed tomography (CT), and MRI allows for accurate and effective diagnosis. The purpose of this review is to describe different non-invasive imaging techniques and highlight their advantages and disadvantages, aiming to provide a guide to clinicians and cardiovascular imaging specialists in choosing the best imaging tools to use in adults with native CoA.

## 1. Introduction

CoA comprises congenital narrowing of the thoracic aorta, most commonly at the isthmus just distal to the left subclavian artery, accounting for ~5–8% of live births with congenital heart disease [1,2]. While often diagnosed in childhood, many patients present in adulthood with hypertension, arm–leg blood pressure differentials, or incidental findings on imaging [1,2,3]. From a life-course perspective, delayed diagnosis may in part reflect challenges in the transition from pediatric to adult congenital heart disease care.

Adult CoA may be native or recurrent after prior repair, carrying significant risks of left ventricular hypertrophy, aortic aneurysm, premature coronary disease, and cerebrovascular events (as intracranial haemorrhage from berry aneurysm) if unmanaged [2,4,5]. Transthoracic echocardiography (TTE) provides initial screening, but its acoustic limitations in adults (particularly pronounced in older adults and obese patients) make cross-sectional imaging—CT and cardiovascular magnetic resonance (CMR)—essential for definitive diagnosis, treatment planning, and surveillance [2,3,6]. These modalities are complementary, not competitive. This review details a modern, integrated imaging strategy, framing CoA as a systemic disease requiring a personalized, lifelong imaging approach to optimize outcomes. Even after anatomically successful repair, patients face a substantial residual cardiovascular risk and excess mortality, highlighting the need for stringent long-term surveillance and more effective preventive strategies [7].

### 1.1. Aortic Coarctation and Associated Cardiovascular Malformations

In adult patients, CoA is seldom an isolated lesion.

CoA may also be associated with other lesions, including a bicuspid aortic valve, sub- or supra-aortic stenosis, mitral valve stenosis, or anomalous origin of right subclavian artery, or may be a cardiac manifestation in some syndromes [4].

The most frequent association is with BAV, present in over 50% of cases [2,8,9]. This association necessitates vigilant, lifelong surveillance of the aortic root and ascending aorta due to the concomitant risks of progressive aortopathy and valvular dysfunction [10], and paediatric cardiology guidelines further recommend early baseline imaging of the entire aorta [11]. Furthermore, the evaluation of aortic regurgitation in a BAV can be challenging due to eccentric jets, underscoring the need for a multimodality imaging approach [10].

Additional congenital cardiac anomalies are common and include ventricular septal defects, persistent ductus arteriosus, and arch hypoplasia [2,4,8]. CoA is also a key component of complex syndromes such as Shone’s complex and hypoplastic left heart syndrome, which in adult populations are encountered almost exclusively among highly selected survivors.

Moreover, there is a well-documented association with specific genetic syndromes. These include Turner syndrome, where CoA and a BAV are prevalent [12], and Loeys–Dietz syndrome, caused by mutations in the TGF-β pathway [13]. Routine genetic testing is not universally recommended for all patients with CoA; rather, a targeted genetic evaluation is generally indicated in the presence of syndromic features, atypical phenotypes, early-onset or aggressive aortopathy, or a suggestive family history [4].

Anomalies of the coronary arteries, such as anomalous origin or course, have also been variably reported in patients with CoA and a BAV [14] (Table 1).

Chronic upstream hypertension induced by CoA provokes profound systemic arterial remodelling, leading to the development of an extensive collateral circulation network via intercostal, internal mammary, and thoracic wall arteries. The presence and degree of these collaterals are highly suggestive on cross-sectional imaging and serve as an important marker of haemodynamic significance, correlating with the chronicity and severity of stenosis [3,15]. The persistent hypertensive state accelerates vascular aging, promoting aortic dilation both proximal and distal to the coarctation and leading to left ventricular concentric hypertrophy. Crucially, these sequelae may endure despite anatomically successful repair, underscoring the necessity for serial assessment of ventricular mass and function to guide ongoing risk stratification and medical therapy [2,5,7,16,17,18].

### 1.2. How It Occurs in Adulthood

Clinical adult presentation depends on the severity of CoA and differs between native and recurrent disease. In contrast to childhood, where coarctation is more often detected due to heart failure, failure to thrive, or a cardiac murmur, adult presentation is typically driven by systemic hypertension, long-standing vascular remodelling, or incidental imaging findings. Native CoA is often suspected during the work-up of secondary hypertension (particularly in adults < 40 years), whereas recurrent CoA is more frequently identified during structured post-repair surveillance or incidentally on cross-sectional imaging [8,19]. Typical symptoms may be caused by pre-stenotic elevated blood pressure such as headache, dizziness, epistaxis, tinnitus, and/or post-stenotic hypoperfusion like claudication, cold feet, and abdominal angina due to the pressure gradient between upper and lower extremities. Non-treated hypertension may also lead to long-term complications like hypertension-mediated organ damage (HMOD), especially left ventricular hypertrophy and/or dysfunction or aortic and intracranial aneurysm, that may result in aneurysmal ruptures, haemorrhages, or dissections [20]. CoA may also be associated with other lesions including a bicuspid aortic valve (50–85%), sub- or supra-aortic stenosis, mitral valve stenosis, and anomalous origin of right subclavian artery or may be a cardiac manifestation in Turner syndrome [4]. Importantly, a non-negligible proportion of adults may be asymptomatic and are diagnosed incidentally when CT/MRI is performed for unrelated indications.

### 1.3. How to Diagnose Aortic Coarctation in Adults

On physical examination, systolic blood pressure is higher in the upper extremities compared with the lower extremities (significant gradient > 20 mmhg). If coarctation is proximal to the left subclavian artery, systolic blood pressure is also higher in the right arm compared with the left arm. Femoral pulses are weaker than brachial and radial pulses. Prominent pulsations and thrill are evident in suprasternal notch. Along the left sternal border and between the scapulae, a systolic–diastolic murmur is present due to collateral circulation [21]. Chest X-ray findings may include double-contouring of the descending aorta due to pre- and post-CoA aortic dilatation and rib notching from the fourth to eight ribs resulting from collateral circulation [22]. Electrocardiography (ECG) may show left ventricular (LV) hypertrophy signs. However, transthoracic echocardiography (TTE) is commonly used as the first-line imaging test for suspected CoA, providing initial anatomic and haemodynamic assessment and post-intervention follow-up; in contemporary adult practice, CT or CMR may also represent the first diagnostic test, particularly when CoA is detected incidentally or when acoustic windows are limited.

### 1.4. How to Treat

Systemic secondary hypertension due to CoA with an arm–leg systolic pressure difference > 20 mmHg or an invasive peak-to-peak gradient ≥ 20 mmHg (CW mean gradient ≥ 22 mmHg), re-coarctation of CoA with ≥20 mmHg, and the presence of signs and symptoms like LV hypertrophy, heart failure, and claudication due to CoA are class I indications for intervention in adults. Significant collateral vessels and lower limb hypoperfusion despite a low gradient (due to possible underestimation) are class II indications for intervention [4]. Endovascular stenting is first-line treatment in adult, and balloon angioplasty is reserved only for re-coarctation [9]; covered stents are preferred to prevent complications (lesions of the aortic wall) in the presence of risk factors such as tortuous aorta, the presence of aneurysms or pseudoaneurysms, or genetically determined aortopathies leading to greater fragility of the wall and a higher risk of dissection [23]. A surgical approach including resection or extended end-to-end anastomosis, patch aortoplasty, or extra-anatomic graft is indicated when a percutaneous approach is not feasible (extensive arch hypoplasia, long-segment disease) or in the presence of concomitant surgical diseases like aortic arch/ascending aorta aneurism, associated significant aortic valve stenosis, or regurgitation [9]. These indications apply to both native and recurrent coarctation in adulthood, integrating symptoms, gradient assessment, and evidence of haemodynamic significance. Long-term series in adults generally show durable gradient relief with a non-zero need for reintervention, most commonly due to restenosis, stent-related complications, or aneurysm surveillance findings, reinforcing the importance of lifelong imaging follow-up [5].

## 2. Diagnosis of Aortic Coarctation: Usefulness and Limitations of Echocardiography

### 2.1. Usefulness and Limitations

Usefulness: TTE is a highly available, low-cost, radiation and contrast-free primary diagnostic and follow-up tool [10]. It allows for anatomical definition and functional evaluation for diagnosis and follow-up after intervention. It provides information about LV size, diastolic and systolic function, and initial myocardial damage by speckle tracking measures. It is the safest method as it does not require either radiation or contrast exposure. Speckle-tracking and deformation indices can detect subclinical myocardial dysfunction and have been reported in adult congenital heart disease cohorts [24]; however, their routine use is not yet universally endorsed in guidelines and should be interpreted in clinical context.

Limitation: The main limitations are the acoustic window with limited visualization of the aortic arch and descending thoracic aorta, operator dependency, doppler gradient inaccuracies due to the presence of collateral circulation, and the absence of extracardiac evaluation of collateral circulation (Table 2). Additionally, doppler-derived gradients are subject to interobserver variability and may underestimate severity when extensive collateral flow reduces the trans-coarctation pressure drop [2].

### 2.2. Anatomical Detail

A suprasternal view allows for evaluation of the aortic arch and isthmus, enabling us to calculate the isthmic diameter-to-ascending aortic diameter ratio, with values < 0.7 suggesting significant narrowing. This index has been mainly derived from paediatric and young adult cohorts and should be interpreted with caution in older adults, as age-related aortic remodelling and measurement variability may influence its accuracy; therefore, it is best used as a supportive rather than standalone diagnostic parameter [2].

From the parasternal long and short axis, it is possible to assess any associated anomalies like BAV, aortic aneurysm, and secondary remodelling like LV hypertrophy.

### 2.3. Functional Data

**Figure 1:**
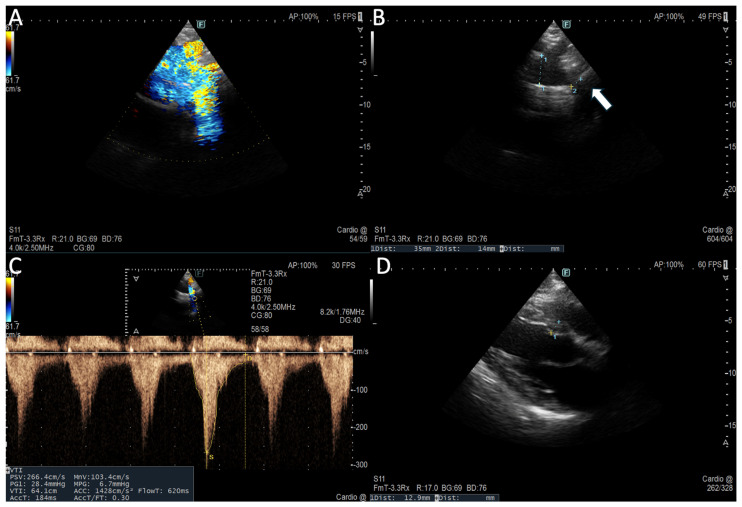
Assessment of aortic coarctation by TTE. (**A**) Aortic arch colour imaging with turbulent flow in suprasternal window. (**B**) Narrowing of isthmus diameter (with arrow) compared to proximal aortic arch (ratio 0.4) in suprasternal window for anatomical assessment. (**C**) Continuous doppler showing peak systolic velocity (>2 m/s) and peak pressure gradient (>20 mmHg) across narrowing site for functional assessment of significant CoA gradient. (**D**) Indirect CoA finding of LV hypertrophic remodelling.

A suprasternal view allows for functional evaluation with continuous-wave doppler (CW) to assess peak systolic velocity (>2 m/s) and peak pressure gradient (>20 mmHg) across the CoA site and pulsed-wave doppler, demonstrating prolonged diastolic flow (diastolic tail) with high velocity (>1 m/s) due to downstream obstruction [25]. A subcostal view shows delayed (>50 ms from ECG R wave) and reduced (<55 cm/s) systolic upstroke velocity and the persistence of anterograde diastolic flow in the distal thoracic and upper abdominal aorta [26] (Figure 1).

Indirect CoA findings may include elevated LV after load with or without ejection fraction reduction [8]. CoA is associated with a higher prevalence of LV diastolic dysfunction and a normal ejection fraction because of the pressure overload that leads to concentric remodelling [26]. Effective repair and systolic pressure control may lead to diastolic function improvement [27].

Speckle-tracking echocardiography is an accurate measure of LV systolic function and subclinical myocardial damage. Early intervention may have positive effects on the preservation of LV systolic function and could improve impaired global longitudinal strain (GLS) [24]. Furthermore, Vivian Wing-yi Li et al. demonstrate that even altered arterial stiffness assessed by TTE is associated with mechanical alterations of LV and left atrium (LA) strain parameters, with an anomalous artery–LV-LA coupling that supports the use of strain indices for the functional surveillance of patients undergoing CoA repair intervention [28].

## 3. Diagnosis of Aortic Coarctation: Usefulness and Limitations of CT Scan

Computed tomography angiography (CTA) is a fundamental tool in the management of adult CoA. Its high spatial resolution provides detailed, three-dimensional visualization of the thoracic aorta, its branch vessels, and the collateral circulation that develops in response to chronic obstruction [1,2,3]. Because many patients require repeated imaging over several decades, cumulative radiation exposure is an important consideration and should be minimized through rigorous dose optimization strategies.

Technical Protocol and Optimization

The diagnostic quality of a CTA study depends on a carefully optimized protocol. To fully evaluate coarctation and the frequently extensive collateral network, the scan must cover the supra-aortic vessels to the diaphragm [3,15]. Technically, thin slices (≤0.625 mm) are required for high-quality multiplanar reconstructions. ECG gating is crucial to eliminate motion in the aortic root and ascending aorta, which is especially important for assessing the associated aortopathy often seen with a bicuspid aortic valve [5,29]. Controlled injection of contrast (typically 1–1.5 mL/kg at 4–6 mL/s), timed from the ascending aorta, is necessary to ensure uniform opacification across the narrowed segment [29,30]. Given that these patients are young and require lifelong imaging, strict dose reduction techniques are mandatory. These include using the lowest possible tube voltage (e.g., 80–100 kVp for normal-weight patients), automatic tube current modulation, and iterative reconstruction algorithms and, when available, dual-energy techniques improve the contrast-to-noise ratio and reduce blooming from calcification or metallic implants [5,29,30] (Table 3).

The analysis should include advanced post-processing: centreline analysis for accurate measurements perpendicular to the vessel lumen, curved planar reformats to visualize the entire arch in a single image, and 3D models that are invaluable for planning procedures and communicating with the heart team [3,30].

### 3.1. Comprehensive Anatomic Assessment and Clinical Implications

Anatomic precision directly impacts clinical decision-making. CTA precisely shows the location and type of narrowing (discrete or long-segment), measures its severity, and identifies post-stenotic dilation [2,3]. The haemodynamic significance can be estimated by calculating the coarctation index (the ratio of the narrowest diameter to the aorta at the diaphragm), with a value below 0.6 suggesting severe disease [2,3]. Beyond the primary lesion, CTA clearly shows arch hypoplasia, anatomic variations (e.g., bovine arch or aberrant subclavian arteries), and the relationship of the lesion to the branch—information that influences treatment decisions [3]. A major strength is its ability to map collateral blood vessels, which are a clear sign of a long-standing, severe blockage [3,15]. Notably, reported thresholds and measurement techniques vary across studies (e.g., reference segment selection and centreline methodology), which can influence reproducibility.

**Figure 2:**
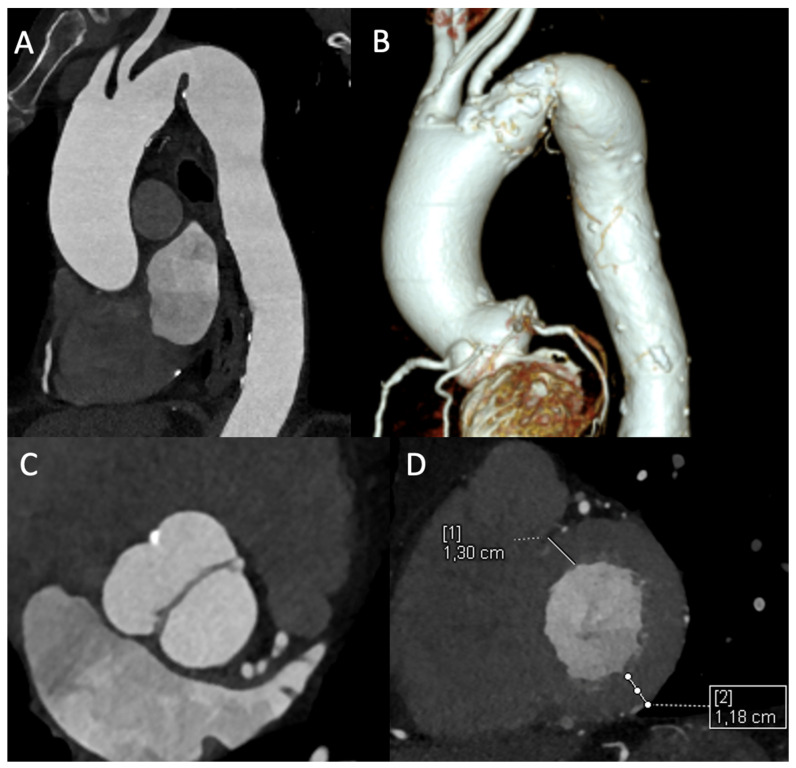
Assessment of aortic coarctation by CT. (**A**) CTA sagittal view of aortic coarctation; (**B**) CTA 3D volume rendering reconstruction of aortic coarctation; (**C**) CTA with detailed view of the bicuspid aortic valve in diastolic phase; (**D**) CTA with reconstruction on ventricular short-axis view and measurement of ventricular walls in diastolic phase for assessment of LV hypertrophy.

Additionally, the examination allows for the assessment of secondary cardiac changes. In the context of chronic pressure overload caused by CoA, the evaluation of left ventricular wall thickness and the presence of concentric hypertrophy are essential. While CMR is the gold standard for volumetric and functional quantification, ECG-gated CTA provides excellent morphological assessment of the ventricles. The detection of significant hypertrophy reinforces the diagnosis of haemodynamically significant and long-standing coarctation, contributing to the overall risk stratification of the patient [5] (Figure 2). While ECG-gated CTA can characterize morphology, quantitative ventricular function and mass assessment remain more accurate and reproducible with CMR.

Emerging technologies such as CT-derived fractional flow reserve (CT-FFR) may help bridge the gap between anatomy and physiology by estimating pressure gradients from standard CTA datasets; however, in aortic coarctation its clinical utility remains investigational and is not yet part of routine decision-making [31].

CTA is particularly valuable for identifying complications that directly affect management decisions. The identification of heavy circumferential calcification at the coarctation site is a critical finding that may contraindicate plain balloon angioplasty due to the risk of aortic rupture, instead favouring primary stenting or surgical repair [3,30]. Similarly, the detection of a calcified patent ductus arteriosus may alter the surgical approach. For aneurysm surveillance after repair, CTA provides the precise measurements needed to determine the timing of intervention [5,29]. The modality is also excellent for characterizing the aortic wall, identifying features like penetrating atherosclerotic ulcers or intramural hematoma that may require specific management [3,5,29]. For patients with a bicuspid aortic valve, an ECG-gated CTA can simultaneously provide accurate measurements of the aortic root and ascending aorta for aortopathy monitoring, making it a comprehensive single-modality assessment for many patients [5,8].

### 3.2. Integration into Therapeutic Decision-Making

The primary clinical value of CTA lies in its ability to guide specific therapeutic choices. In transcatheter stenting planning, CTA provides more than just anatomic measurements; it offers prognostic information that directly influences device selection and procedure strategy. The precise characterization of landing zones helps select appropriate stent sizes to prevent complications like stent migration or aortic wall injury [1,3,30]. The relationship of the coarctation to the left subclavian artery determines whether coverage is feasible or if surgical alternatives should be considered.

For surgical planning, especially in re-operations, CTA provides essential information for risk stratification. The identification of large, fragile collateral vessels alerts the surgical team to potential bleeding risks and may influence the choice of surgical approach [2,3]. The relationship between the sternum and major vascular structures is crucial for planning safe re-entry in re-do operations. In patients with arch hypoplasia, CTA measurements help determine whether a simple resection or extended arch repair is necessary [2,3].

Unlike paediatric practice, vessel growth considerations are generally not relevant in adults; planning focuses on landing zones, wall integrity, and long-term surveillance of repair sites.

After intervention, CTA is indispensable for surveillance. The use of sharp reconstruction kernels minimizes blooming artifacts, allowing for accurate assessment of stent apposition and detecting in-stent restenosis or pseudoaneurysm formation at surgical anastomoses [1,3,29].

A limitation is that subtle early neointimal proliferation may be difficult to detect depending on stent type and reconstruction parameters, and should be interpreted alongside clinical and haemodynamic data.

### 3.3. Usefulness and Limitations

Usefulness: The primary strength of CTA lies in its unparalleled anatomic definition. It is essential for confirming the diagnosis, planning interventions (both transcatheter and surgical), and long-term surveillance, especially in stented patients where it produces minimal artifacts [29]. Its ability to precisely characterize the coarctation (location, length, severity, calcification), map the collateral circulation, and evaluate the entire thoracic aorta and branch vessels makes it the cornerstone of pre-procedural planning [2,3,15]. Furthermore, ECG-gated CTA allows for concurrent assessment of the aortic root for associated aortopathy and provides a morphological evaluation of left ventricular hypertrophy, adding functional context to the anatomic data [5,8]. Its wide availability and rapid acquisition also make it the preferred test in urgent settings or when CMR is contraindicated.

Limitations: The principal limitation of CTA is its inability to directly quantify haemodynamics; it cannot measure pressure gradients across the stenosis, which require doppler ultrasound, CMR, or catheterization [1,2,3]. Furthermore, the cumulative lifetime risk from ionizing radiation must be actively managed with strict dose reduction protocols [5,30]. The necessity for iodinated contrast media carries a risk of nephrotoxicity and allergic reactions, mandating careful patient selection, pre-procedural hydration in at-risk individuals, or consideration of alternative imaging in cases of severe renal impairment [5,29]. Typical effective doses for modern thoracic CTA protocols are in the single-digit mSv range, but may vary widely with patient size and gating; documenting dose and applying ALARA principles are recommended for lifelong follow-up (Table 4).

## 4. Diagnosis of Aortic Coarctation: Usefulness and Limitations of MRI

CMR is a pivotal non-invasive modality for the comprehensive management of adults with CoA, uniquely providing a *one-stop-shop* evaluation that integrates detailed anatomy, haemodynamic significance, and end-organ (ventricular) consequences—all without ionizing radiation [1,2,5,8,10].

**Figure 3:**
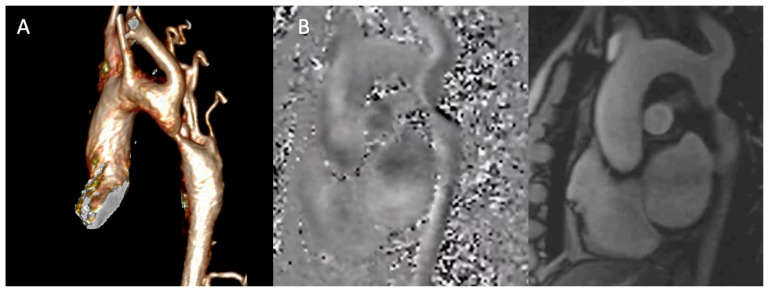
(**A**). CE-MRA 3D volume rendering reconstruction of aortic coarctation. (**B**) Phase contrast MRI in sagittal view of aorta.

A tailored CMR protocol for adult CoA is systematic, addressing the multifocal nature of the disease through five core components designed to answer specific clinical questions [1,8,10,32]:

1. **Cine Imaging for Anatomy and Function:** Balanced steady-state free precession (bSSFP) sequences are fundamental. Beyond standard long- and short-axis planes for the quantitative assessment of LV volumes, ejection fraction, and mass—the non-invasive gold standard—specific cine images aligned along the double-oblique axis of the aortic arch are crucial. These “aortic cines” dynamically visualize the jet across the coarctation and any associated arch hypoplasia [10,32]. Furthermore, high-resolution cine images acquired in the short-axis plane of the aortic valve allow for a precise anatomical assessment of valve morphology, enabling differentiation between tricuspid and BAV. The same views provide a functional assessment, revealing restricted leaflet motion suggestive of stenosis or a central coaptation defect indicative of regurgitation. This evaluation is a critical part of the examination, given the high prevalence of BAV and concomitant valvulopathy in the CoA population.
2. **Angiography for Road Mapping:** Contrast-enhanced magnetic resonance angiography (CE-MRA) provides high-resolution 3D anatomy (Figure 3A). Acquired with isotropic voxels (≤1.5 mm), these datasets allow for exquisite multiplanar reformations, precisely defining the coarctation’s location, length, and minimal diameter. For patients where gadolinium is contraindicated, non-contrast 3D acquisitions (e.g., respiratory-navigated, ECG-gated bSSFP) offer a reliable alternative [3,8,32]. Furthermore, modern non-contrast techniques like 3D Dixon-based water–fat separation provide robust luminal and wall characterization, simultaneously visualizing the aortic lumen and periaortic fat, which can be valuable in post-surgical cases.
3. **Phase-Contrast MRI (**Figure 3**B):** Through-plane phase-contrast sequences, with the imaging slice positioned perpendicular to the aorta at the stenosis, allow for estimation of the peak instantaneous gradient (ΔP ≈ 4 V^2^max). Careful slice placement is critical, as misalignment can significantly underestimate the velocity. Crucially, flow measurements in the ascending aorta and at the diaphragm enable calculation of the collateral flow index: (Distal Aortic Flow—Ascending Aortic Flow)/Ascending Aortic Flow. An index >10–20% provides objective, quantitative evidence of haemodynamically significant stenosis, which is critical for advocating intervention in asymptomatic patients [16,32]. This is particularly useful in asymptomatic patients with borderline doppler gradients, where collateral flow quantification may better reflect true haemodynamic burden.
4. **Myocardial Tissue Characterization:** Late gadolinium enhancement (LGE) imaging can identify focal myocardial fibrosis. Native T1 mapping and an extracellular volume (ECV) fraction offer a quantitative assessment of diffuse interstitial fibrosis, providing a sensitive marker of preclinical myocardial disease that may influence medical therapy [8,10]. While still limited as a research tool in this specific population, the presence of elevated ECV may identify a subgroup of patients with more advanced hypertensive heart disease who warrant closer monitoring [33].
5. **4D Flow:** Emerging 4D flow MRI techniques enable comprehensive, three-dimensional visualization of blood flow patterns, wall shear stress, and energy loss throughout the thoracic aorta. This allows for the quantification of regional wall shear stress in the ascending aorta, which may help stratify the risk of progressive aortopathy in patients with an associated bicuspid aortic valve, moving beyond simple diameter measurements for risk assessment [34]. At present, adoption is limited by availability, acquisition/processing time, and incomplete inter-vendor standardization, and therefore remains largely confined to expert centres and research workflows.

**Figure 4:**
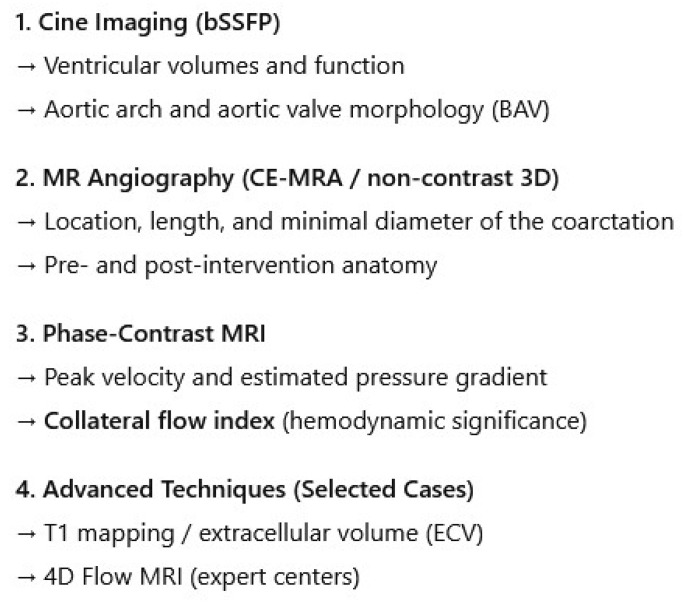
Cardiovascular magnetic resonance (CMR) workflow for adult aortic coarctation.

For clarity, a schematic workflow summarizing sequence selection and the corresponding clinical objectives is provided in Figure 4.

### Usefulness and Limitations

Usefulness: CMR is the preferred modality for initial diagnosis and serial follow-up in young, non-stented patients due to its lack of radiation. Its ability to provide a synergistic assessment of anatomy, ventricular impact, and haemodynamics aligns perfectly with the understanding of CoA as a complex systemic disease and provides robust risk stratification. A key advantage is its capacity for quantitative haemodynamic assessment. The collateral flow index, in particular, is a unique strength, offering a direct measure of haemodynamic significance that is often more sensitive than anatomic measurements alone and is crucial for risk stratification [5,9,16]. The clinical utility of CMR is profound at every stage. In the newly diagnosed adult, it serves as a definitive baseline examination. This dataset is indispensable for deciding if and when intervention is warranted [1,2,10]; for example, a patient with discrete narrowing but a high collateral flow index and significant LV hypertrophy has a clear physiological indication for repair, even in the absence of severe symptoms [16]. In post-intervention surveillance, CMR is the modality of choice. It effectively monitors for re-coarctation and, most importantly, tracks the regression of LV mass—a key marker of successful treatment and reverse remodelling. The persistence of LV hypertrophy despite a seemingly adequate anatomical repair is a poor prognostic sign and should prompt aggressive medical management [17,18]. Looking ahead, the integration of artificial intelligence for automated quantification of ventricular parameters and aortic dimensions will further enhance the reproducibility and efficiency of CMR. Furthermore, the ongoing validation of 4D flow-derived biomarkers, such as energy loss and wall shear stress, promises a future where CMR can not only diagnose but also better predict an individual patient’s risk of disease progression.

Limitations: Acknowledging CMR’s limitations is essential for its appropriate application. Metallic implants, particularly older stents, can cause significant susceptibility artifacts, obscuring the lumen. In such cases, CTA is the complementary modality of choice for definitive anatomic evaluation [3,5,29]. Turbulent flow can lead to signal loss and potential underestimation of peak velocities; careful technique is required. It is also important to recognize that in the presence of significant arrhythmia, obtaining diagnostic image quality can be challenging, though newer arrhythmia rejection algorithms are mitigating this limitation [35]. The examination has a longer acquisition time and is not as widely available as CTA. It is also contraindicated in patients with certain non-MRI-compatible implants and severe claustrophobia (Table 5).

## 5. Conclusions

Adult CoA is a lifelong systemic disease, not merely focal aortic stenosis. Optimal long-term outcomes, therefore, depend on an integrated, multimodal imaging strategy that is dynamically tailored to the individual patient. This approach strategically leverages the specific strengths of each technique: echocardiography serves as the essential first-line tool for initial screening, functional valvular assessment, and basic ventricular evaluation; CTA provides unmatched spatial resolution for definitive anatomic diagnosis, procedural planning of interventions, and surveillance of stents or complex anatomy; and CMR offers a comprehensive, radiation-free “one-stop-shop” for precise quantification of haemodynamic significance, collateral flow, and serial monitoring of ventricular remodelling.

This synergistic, patient-centred strategy allows for sophisticated risk stratification—identifying patients with persistent collateral flow or significant LV hypertrophy who may benefit from intensified therapy—and enables the design of personalized surveillance intervals. Notably, the persistence of left ventricular hypertrophy despite anatomically successful repair represents a key prognostic marker, reflecting residual haemodynamic burden and long-standing vascular remodelling, and should prompt closer clinical and imaging surveillance as well as aggressive optimization of medical therapy [17,18]. Rigorous blood pressure control combined with this standardized, expert-centred imaging protocol is essential to mitigate late complications. Future integration of advanced techniques like 4D flow MRI and artificial intelligence for automated measurements promises to further refine risk prediction and personalize long-term management, translating anatomic correction into durable cardiovascular health across the adult lifespan. For example, a typical strategy may include annual clinical assessment and blood pressure evaluation, with cross-sectional imaging (CMR or CTA depending on repair type and implants) every 3–5 years, and shorter intervals when residual gradients, aneurysms, or persistent LV hypertrophy are present.

The novel perspective of this review lies in moving beyond a simple comparison of imaging techniques to frame this dynamic, integrated strategy for lifelong care. The clinical implications are clear: imaging must be comprehensive, reflecting the complex nature of CoA as a disease of the aorta and myocardium; a tailored approach leveraging the complementary strengths of echocardiography, CTA, and CMR is essential to guide intervention and optimize outcomes; and this multimodal framework facilitates personalized surveillance to mitigate late risks such as aneurysm formation, re-coarctation, and hypertensive heart disease. In contrast to prior reviews that primarily focus on a single modality’s strengths and limitations, we emphasize a dynamic, patient-tailored framework that integrates modality choice with disease chronicity, repair status, and evolving lifetime risk.

## Figures and Tables

**Table 1:** Frequency of cardiovascular malformations associated with CoA.

Associated Malformation/Condition	Reported Frequency in Adults with CoA *
Bicuspid aortic valve (BAV)	>50% (up to 60–70% in some cohorts)
Subvalvular or supravalvular aortic stenosis	~5–10%
Ventricular septal defect (VSD)	~5–20%
Patent ductus arteriosus (PDA)	~5–10% (more common in paediatric populations)
Aortic arch hypoplasia	~10–30%
Congenital mitral valve disease/mitral stenosis	<5–10%
Anomalous origin of the right subclavian artery	~3–5%
Shone’s complex	Rare (<5% of adults with CoA)
Hypoplastic left heart syndrome (adult survivors)	Very rare (highly selected cohort)
Turner syndrome (association with CoA ± BAV)	CoA in ~10–20% of patients; BAV in up to 30–40%
Loeys–Dietz syndrome	Variable; CoA reported in selected subgroups
Coronary artery anomalies (abnormal origin or course)	Variable, ~2–5%

* Frequencies are approximate and vary according to patient age, study population (paediatric vs. adult), and cohort type (surgical, outpatient, or imaging-based).

**Table 2:** TTE in adult aortic coarctation: usefulness and limitations.

Aspect	Usefulness	Limitations
Anatomic Detail	Anatomical assessment of aortic arch and isthmus	Operator and window dependency; aorta in toto not available
Speed and Access	Cheap, rapid, and widely available	Execution speed depends on availability/difficulty of finding a good window
Functional Data	Direct flow/pressure quantification; functional LV assessment	Collateral circulation not available
Safety Profile	No radiation and no contrast exposure	

**Table 3:** CTA protocol acquisition for aortic coarctation study.

CTA Protocol Element (Adult CoA)	Practical Note
Coverage	From supra-aortic vessels to diaphragm to assess CoA, arch, and collaterals.
Acquisition	Thin slices (≤0.625 mm) with multiplanar/3D reconstructions.
ECG gating	Recommended for root/ascending aorta and valve/aortopathy assessment.
Contrast	Weight-based (≈1–1.5 mL/kg) at 4–6 mL/s; bolus timing in ascending aorta.
Dose reduction	Low kVp (e.g., 80–100 in normal-weight), tube modulation, and iterative reconstruction; consider dual-energy when available.
Post-processing	Centreline analysis + curved planar reformats + 3D VR for planning.

**Table 4:** CT in adult aortic coarctation: usefulness and limitations.

Aspect	Usefulness	Limitations
Anatomic Detail	High spatial resolution; excellent 3D reconstructions; robust stent visualization.	Blooming from calcification/metal; motion artifact if ungated.
Speed and Access	Rapid acquisition; widely available.	Ionizing radiation; cumulative exposure risk.
Functional Data	Optional cine for LV volumes if ECG-gated.	No direct flow/pressure quantification.
Safety Profile	Unaffected by MRI-incompatible devices.	Iodinated contrast risks (nephrotoxicity, allergy).

**Table 5:** MRI in adult aortic coarctation: usefulness and limitations.

Aspect	Usefulness	Limitations
Global Assessment	Single exam for anatomy, flow, and ventricular function; no ionizing radiation; excellent tissue characterization	Longer acquisition time; breath-hold dependence; contraindications (non-compatible implants, claustrophobia)
Haemodynamics	Accurate phase-contrast gradients; quantitative collateral flow assessment; 4D flow potential for advanced profiling (WSS, energy loss)	Signal loss in turbulent flow may lead to underestimation of peak velocities; artifacts from metallic stents/coils may obscure the lumen
Aortopathy and Valvular Function	Reproducible aortic diameters; comprehensive anatomical and functional valve assessment (e.g., BAV, stenosis, regurgitation)	Gadolinium contrast contraindicated in severe renal failure
Clinical Indications and Surveillance	**Primary Indication:** Comprehensive initial evaluation and routine surveillance in non-stented patients; ideal for serial follow-up in young patients; monitoring of ventricular reverse remodelling	CTA is preferred for surveillance after stenting and for definitive anatomic definition in cases of ambiguous MRI findings

## Data Availability

No new date were created or analyzed in this study. Data sharing is not applicable to this article.

## Abbreviations

The following abbreviations are used in this manuscript:

BAV	bicuspid aortic valve
CoA	aortic coarctation
TTE	transthoracic echocardiography
CT	computed tomography
CMR	cardiovascular magnetic resonance
HMOD	hypertension-mediated organ damage
ECG	electrocardiography
LV	left ventricle
GLS	global longitudinal strain
LA	left atrium
CTA	computed tomography angiography
CT-FFR	CT-derived fractional flow reserve
CE-MRA	contrast-enhanced magnetic resonance angiography
LGE	late gadolinium enhancement
ECV	extracellular volume
